# 

**Published:** 1886-03

**Authors:** 


					﻿Whole Number, 315.
MARCH, I886.
Vol. Lit., Number 3.
the
CHICAGOTOimOURNAL AND EXAMHO
EDITORS:
S. J. JONES, M.D, LL.D.
W. W. JAGGARD, A.M., M.D.
HAROLD N. MOYER, M.D.
PUBLISHED MONTHLY by
CBARK & BOKGLEY,
Under direction of
THE CHICAGO MEDICAL PRESS ASSOCIATION,
163-165 Dearborn Street.
				

